# Coral calcification in a changing World and the interactive dynamics of pH and DIC upregulation

**DOI:** 10.1038/ncomms15686

**Published:** 2017-05-30

**Authors:** Malcolm T. McCulloch, Juan Pablo D’Olivo, James Falter, Michael Holcomb, Julie A. Trotter

**Affiliations:** 1 Oceans Institute and School of Earth Sciences, The University of Western Australia, Crawley, Western Australia 6009, Australia; 2 ARC Centre of Excellence for Coral Reef Studies, The University of Western Australia, Crawley, Western Australia 6009, Australia

## Abstract

Coral calcification is dependent on the mutualistic partnership between endosymbiotic zooxanthellae and the coral host. Here, using newly developed geochemical proxies (δ^11^B and B/Ca), we show that *Porites* corals from natural reef environments exhibit a close (*r*^2^ ∼0.9) antithetic relationship between dissolved inorganic carbon (DIC) and pH of the corals’ calcifying fluid (cf). The highest DIC_cf_ (∼ × 3.2 seawater) is found during summer, consistent with thermal/light enhancement of metabolically (zooxanthellae) derived carbon, while the highest pH_cf_ (∼8.5) occurs in winter during periods of low DIC_cf_ (∼ × 2 seawater). These opposing changes in DIC_cf_ and pH_cf_ are shown to maintain oversaturated but stable levels of carbonate saturation (*Ω*_cf_ ∼ × 5 seawater), the key parameter controlling coral calcification. These findings are in marked contrast to artificial experiments and show that pH_cf_ upregulation occurs largely independent of changes in seawater carbonate chemistry, and hence ocean acidification, but is highly vulnerable to thermally induced stress from global warming.

## Abstract (web summary)

Coral reefs are biologically diverse and productive ecosystems, yet our understanding of the impacts of ocean acidification on biocalcification is limited. Here, the authors show that pH upregulation and the biological control of dissolved inorganic carbon in calcifying fluids of *Porites* corals are linked.

Scleractinian corals together with their endosymbiotic dinoflagellates, *Symbiodinium* (zooxanthellae), have been spectacularly successful in building the tropical coral reef edifices that dominate many shallow-water environments and harbour more than one-third of the oceans’ biodiversity. The ongoing viability of these iconic^1^ tropical reef systems is however in question^2^,^3^, with symbiont-bearing shallow-water corals now facing the combined challenge of both global warming and ocean acidification from rapidly rising levels of CO_2_ (ref. 4). Critical to the success of reef-building corals is their ability to extract dissolved inorganic carbon (DIC) from seawater and efficiently convert it into calcium carbonate, the major constituent of their skeletons. While much progress has been made in identifying many of the key elements of the biologic machinery that are integral to the biocalcification process^5^,^6^,^7^ (Fig. 1), there are still significant gaps in our understanding. Foremost is the relationship between declining seawater pH and its impact on pH upregulation of the coral’s extracellular calcifying fluid^8^,^9^,^10^, a process that occurs at least in part via Ca-ATPase pumping of Ca^2+^ ions into the calcifying region in exchange for the removal of protons^11^. Of equal but largely overlooked importance, are the mechanisms via which the various pH-dependent species of DIC (that is, CO_2_, HCO_3_^−^ or CO_3_^2−^) are produced, transported, and then inter-converted at the site of calcification. It has also long been recognized^12^,^13^ that light plays a key role in driving rates of calcification, and that light-enhanced calcification occurs as a result of the photosynthetic activity of endosymbiont dinoflagellates (zooxanthellae), providing both energy and additional carbon needed to drive calcification. The exact mechanism(s) by which coral calcification is linked to endosymbiont photosynthesis has, however, remained largely enigmatic at the polyp scale (Fig. 1) the zooxanthellae are physically separated from the site of calcification^13^,^14^,^15^ and, apart from pH, few direct measurements exist^16^ of the chemical conditions necessary to constrain the biocalcification process. Here we provide new evidence for an intimate link between the biologically mediated process of pH_cf_ upregulation of the calcifying fluid and biological control over the concentration of DIC in the calcifying fluid (DIC_cf_). We find that over annual timescales there is an inverse correlation between pH_cf_ and DIC_cf_. This acts to maintain relatively stable levels of aragonite saturation in the calcifying fluid, and hence near-optimal rates of coral calcification, despite large seasonally driven variations in metabolically supplied DIC.

## Results

### Reef-water and coral calcifying fluid carbonate chemistry

To reconstruct the carbonate chemistry of the calcifying fluid from which corals precipitate their aragonite skeleton, we use the boron isotopic composition (δ^11^B) as a proxy for the calcifying fluid pH_cf_ (refs 10, 17, 18). For determining the carbonate ion concentrations [CO_3_^2−^]_cf_ in the calcifying fluid, we use the combined δ^11^B-B/Ca proxy^19^. The application of the δ^11^B-B/Ca carbonate ion proxy has now been made possible by recent experimental measurements of the B/Ca carbonate ion distribution coefficient^19^, a major limitation of previous studies^20^ (see ‘Methods’ section). To examine how the chemistry of the calcifying fluid varies seasonally under ‘real-world’ reef conditions, we have analysed the skeletons of massive *Porites* collected from Davies Reef in the Great Barrier Reef and from Coral Bay Ningaloo Reef for which reef-water pH and sea-surface temperatures (SST) records are available^21^,^22^ (see ‘Methods’ section). Species of massive *Porites* coral are ideal for reconstructing seasonal changes in the composition of their calcifying fluid since they are long-lived and, more importantly, the architecture of their skeleton has a relatively straightforward chronology that facilitates well-constrained timing of their skeletal growth at seasonal resolution^23^. Given that only limited records of seasonal changes in local seawater carbonate chemistry are available^22^,^24^, these data are supplemented by model estimates^24^ of the reef-induced pH variability. The Great Barrier Reef and Ningaloo Reef sites (see ‘Methods’ section) have a typical seasonal range in temperature from ∼23 to 28 °C, as well as relatively narrow seasonal ranges in seawater pH_sw_ (total scale) from ∼8.02 in summer to ∼8.08 in winter (Fig. 2). This limited seasonal range in average reef-water pH_sw_ of ∼0.06 pH units is comparable to that observed in the open ocean^25^, a reflection of the tight balance between production and respiration^24^ combined with the limited residence time of waters in most wave and tidally driven reef systems^21^.

### Covariation of calcifying fluid pH_cf_ and DIC_cf_

In contrast to the limited variation in reef-water pH_sw_, we find that *Porites* colonies from both Davies Reef and Coral Bay exhibit strong seasonal changes in pH_cf_, from ∼8.3 during summer to ∼8.5 during winter (Fig. 2). This represents an elevation in pH_cf_ relative to ambient seawater of ∼0.4 pH units together with a relatively large seasonal range in pH_cf_ of ∼0.2 units. These observations are in stark contrast to the far more muted changes based on laboratory-controlled experiments^9^,^17^. These inferred laboratory responses^10^ in calcifying fluid pH (pH*_cf_) are shown in Figs 2 and 4, where the expected seasonal range is ∼0.02 pH units, an order of magnitude smaller than those actually observed in reef environments. The explanation for this unexpectedly large range in seasonal pH_cf_ present under natural reef conditions becomes apparent from the exceptionally strong and inverse correlations between pH_cf_ and DIC_cf_ (*r*^2^=0.88–0.94) present at the colony level (Fig. 3a,b).

Here DIC_cf_ reaches its highest values in summer (× 2.0 to × 3.2 higher than ambient seawater) and lowest values in winter, whereas pH_cf_ shows the opposite pattern. This seasonal variability in DIC_cf_ is consistent with light- and temperature-driven changes in the supply of metabolic DIC provided by the endosymbionts and/or the bicarbonate anion transporters within the coral host^7^. Thus, each *Porites* colony forms a distinctive subparallel array characterized by a distinctive range in DIC_cf_ that is inversely correlated to pH_cf_. Since the concentrations of carbonate ion [CO_3_^2−^] and consequently the aragonite saturation state (*Ω*_cf_=[Ca^2+^]_cf_ [CO_3_^2−^]_cf_/*K*_arag_) of the calcifying fluid increases with increasing DIC_cf_ and pH_cf_, the observed antithetic seasonal changes in these parameters results in a more muted seasonal variation in *Ω*_cf_ (±5% to ±10%, Fig. 3a) compared to that expected from changes in only pH_cf_ (±30%) or DIC_cf_ (±12%) acting alone. While there remains a subdued positive correlation of *Ω*_cf_ with temperature (Fig. 3a), the inverse correlations between pH_cf_ and DIC_cf_ (Fig. 2a,b) indicate that the coral is actively maintaining both high (∼ × 4 to × 6 seawater) and relatively stable (within ±10% of mean) levels of elevated *Ω*_cf_ year-round.

While the absolute levels of enhanced *Ω*_cf_ are not dissimilar to previous qualitative estimates^17^,^26^, the finding of significantly higher but relatively limited ranges in DIC_cf_ of ∼ × 2.0 to ∼ × 3.2 seawater, is not generally consistent with recent micro-sensor^16^ measurements. This difference may reflect the intrinsic limitations^6^ of using probes that are 15–20 μm wide to measure the chemistry within the much narrower and irregular (1–10 μm) calcifying region. Additionally, separate probes are required for measurements of pH_cf_ and [CO_3_^2−^]_cf_, introducing further uncertainty, likely accounting for the large variability of *in situ* measured CO_3_^2−^ and hence inferred DIC_cf_ (∼ × 1.4 to × 4.2 seawater). Finally and most importantly, regardless of the method employed, we find that measurements conducted under controlled, static, laboratory conditions^10^ are unlikely to be representative of natural reef conditions due to the interactive dynamics of pH_cf_ and DIC_cf_ upregulation described herein.

## Discussion

The underlying reason for the dynamic, antiphase relationship between pH_cf_ versus DIC_cf_, can be explained by the ability of the coral to ‘control’ what is arguably^27^ one of its most fundamental physiological processes, the growth of its skeleton within which it lives. For example, during winter (Fig. 2), there is a large systematic decrease in the abundance of metabolic DIC (∼25%), presumably as a consequence of reductions in both light and temperature. Since higher pH shifts the carbonate equilibria to favour CO_3_^2−^ relative to HCO_3_^−^, the greater increase in pH_cf_ in winter (∼8.5) compared to summer (∼8.3) increases the concentration of carbonate ions within the calcifying fluid (and therefore *Ω*_cf_) for the same DIC_cf_. This increase in winter pH_cf_ therefore partially counters the seasonal slowdown in host-symbiont carbon metabolism. Hence during the cooler periods, higher pH_cf_ enhances *Ω*_cf_ and hence partially mitigates the reduced temperature-dependent kinetics of calcification because rates of mineral precipitation are proportional to (*Ω*−1)^*n*^, where *n* is the temperature-dependent order of the reaction^28^ (*n*=1.3–2.0 for most reef habitats). During summer, the opposite behaviour is observed, with higher rates of metabolic DIC_cf_ partially offset by decreases in pH_cf_, resulting in a concomitant decrease in the carbonate saturation state of the calcifying fluid (*Ω*_cf_) and hence moderated (albeit still high) rates of calcification (Fig. 4c,d).

This implies that during summer, zooxanthellae-derived DIC_cf_ is being supplied in excess of the ‘optimal’ requirements for the biologically mediated process of skeleton building. Thus, while existing mineral rate kinetics indicate that rates of calcification are still a factor of two- to fourfold higher in summer than in winter, this range is significantly less than the estimated eightfold higher summer rates (Fig. 4c,d) if constant levels of elevated pH_cf_ upregulation were operative, as implied from the artificial constant seawater pH_sw_ and temperature experiments^10^.

Although our findings are based only on species of *Porites* from the Pacific and Indian Oceans, they nevertheless have important implications for our understanding of how reef-building corals in general will respond to climate change. The occurrence, for example, of the highest pH_cf_ values during winter, when metabolically derived sources of energy are at a minimum, provides further evidence against the proposition that pH_cf_ upregulation is an energetically costly process^29^, and will therefore decline as seawater pH_sw_ decreases due to ocean acidification. This is supported by results of the free ocean carbon enrichment experiment^30^ conducted within the GBR Heron Island lagoon, where corals subjected to both natural and superimposed fluctuations in seawater pH_sw_ exhibited essentially constant pH_cf_ upregulation, a condition referred to by those authors^30^ as ‘pH homoeostasis’. These findings, combined with measurements of even higher pH_cf_ in azooxanthellate deep-sea corals^31^ (pH_cf_ >8.6), are thus consistent with inferences that Ca-ATPase-driven pH_cf_ upregulation is a relatively energetically inexpensive process^17^. These observations, in conjunction with the highly correlated and anti-cyclical seasonal changes in both pH_cf_ and DIC_cf_, therefore argue against the reduction of pH_cf_ in summer being a result of the passive feedback from higher rates of calcification producing more protons thereby lowering pH_cf_. Thus, while this possibility cannot yet be entirely excluded, the higher production rates of zooxanthellae-derived metabolites that are presumably available in the summer to facilitate enhanced Ca-ATPase activity, also suggest that the lower summer levels of pH_cf_ is not due to intrinsic limitations in the Ca-ATPase H^+^ pumping, but rather physiological controls on growth rate. Furthermore, similar anti-correlated changes in pH_cf_ and DIC_cf_ are present in *Porites* from both Davies and Ningaloo Reefs, despite large differences in growth rates.

**Figure 1:**
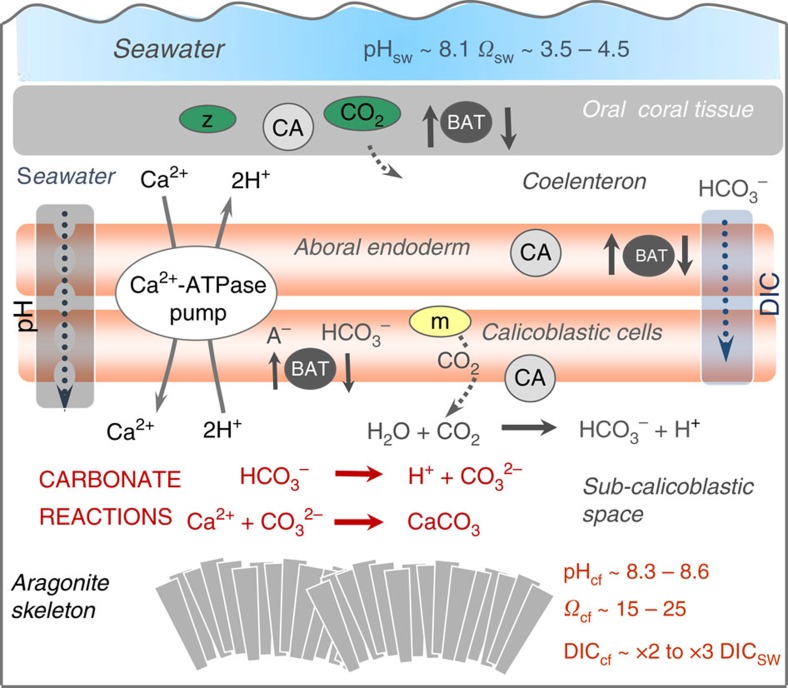
Mechanisms involved in coral calcification. Calcification occurs within the subcalicoblastic space from an initial seawater-derived fluid with additional metabolic sourced supply of DIC^5^,^6^,^7^. Elevation of calcifying fluid pH_cf_ occurs via removal of protons from the calcification site by Ca^2+^-ATPase exchangers. The carbonic anhydrases (CA) catalyse the forward reactions converting CO_2_ into HCO_3_^−^ ions^7^,^34^. Transfer of DIC into the subcalicoblastic space may occur via diffusion of CO_2_ and/or by HCO_3_^−^ pumping via bicarbonate anion transporters (BAT)^5^,^6^,^7^. The link between the activity of zooxanthellae located in the oral coral endoderm tissue to the generation of metabolic DIC within the aboral endoderm and calicoblastic cells (orange) and transport to the calcifying fluid remains uncertain^5^,^6^,^7^ (Figure modified from McCulloch *et al*.^31^).

**Figure 2:**
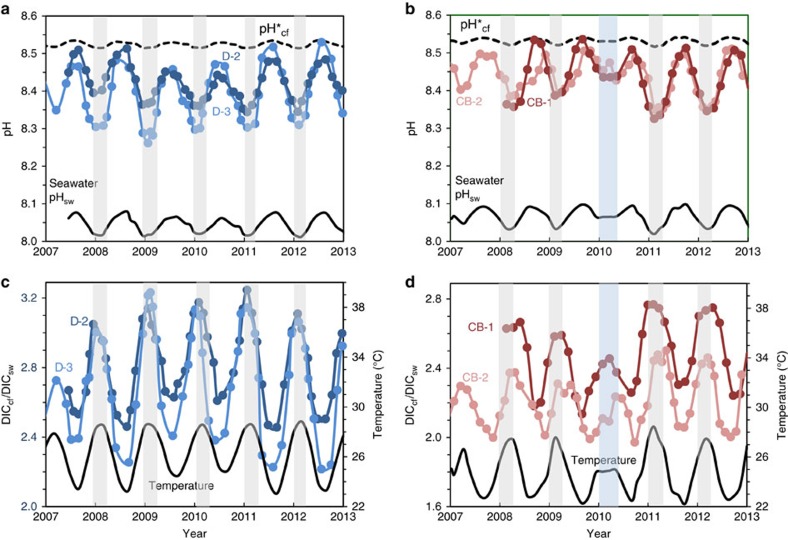
Seasonal time series of coral calcifying fluid pH_cf_ and DIC_cf_. (**a**) *Porites* spp. coral calcifying fluid pH_cf_ derived from δ^11^B systematics (see ‘Methods’ section and Supplementary Data) for colonies D-2 and D-3 from Davies Reef (18.8° S) in the Great Barrier Reef, Queensland. Shading denotes the summer period when pH_cf_ and seawater pH_sw_ values are at a minimum. Dashed line shows pH*_cf_ expected from artificial experimental calibrations (pH*_cf_=0.32 pH_sw_+5.2)^10^,^17^ with an order of magnitude lower seasonal range than measured pH_cf_ values. (**b**) Same as previous for *Porites* colonies from Coral Bay (CB-1 and CB-2) in the Ningaloo Reef of Western Australia (23.2° S) showing seasonal fluctuations in pH_cf_ and seawater pH_sw_. The blue shading denotes the anomalously cool summer temperatures in 2010. (**c**) Enrichments in calcifying fluid DIC_cf_ (left axis; coloured circles) derived from combined B/Ca and δ^11^B systematics together with synchronous seasonal variations in reef-water temperatures (right axis; black line) for *Porites* colonies from Davies Reef (GBR). The strong temperature/light control on DIC_cf_ is consistent with enhanced metabolic activity of zooxanthellae symbionts in summer. (**d**) Same as previous but for *Porites* from Coral Bay (Ningaloo Reef, Western Australia).

**Figure 3:**
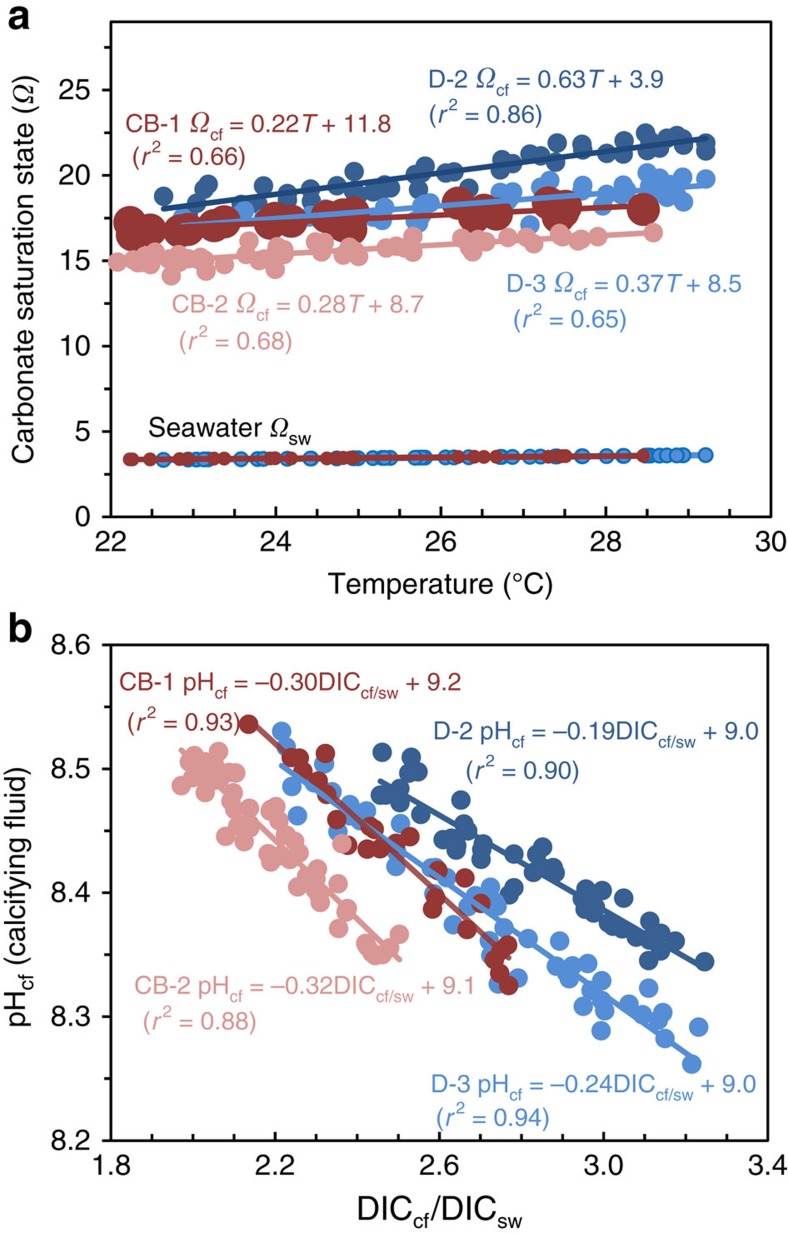
Covariation between calcifying fluid parameters *Ω*_cf_ versus seasonal temperature and pH_cf_ versus DIC_cf_. (**a**) Covariation of calcifying fluid saturation state (*Ω*_cf_) with reef-water temperature showing a five- to sixfold elevation in *Ω*_cf_ relative to reef-waters for *Porites* corals from Davies Reef (D-2 and D-3) in the Great Barrier Reef and from Coral Bay (CB-1 and CB-2) in the Ningaloo Reef. Note the very narrow range (±5 to ±10%) of high *Ω*_cf_ values for each colony. (**b**) Subparallel arrays of inversely correlated (*r*^2^=0.88–0.94) calcifying fluid pH_cf_ and DIC_cf_/DIC_sw_ values reflecting specific bio-environmental controls at the colony level on metabolic DIC_cf_/DIC_sw_. Seasonal variations in metabolic supplied DIC_cf_ are offset by opposing changes in pH_cf_ that act to moderate the overall variations in *Ω*_cf_, the ultimate controller of skeletal growth rates.

**Figure 4:**
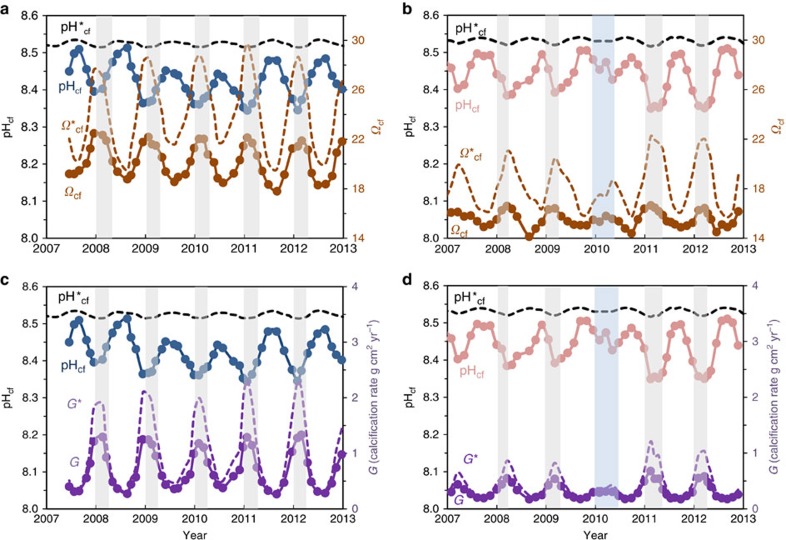
Seasonal time series of calcifying fluid pH_cf_ and *Ω*_cf_ together with calculated calcification rates G. (**a**) Calcifying fluid pH_cf_ and *Ω*_cf_ values for *Porites* coral (D-2) from Davies Reef (GBR), where *Ω*_cf_=[Ca^2+^]_cf_ [CO_3_^2−^]_cf_/*K*_arag_. Dashed line shows the Ω*_cf_ calculated using fixed experimental^10^,^17^ pH*_cf_ values (see Fig. 2a,b). (**b**) Same as previous for Coral Bay (Ningaloo Reef, Western Australia) *Porites* (CB-2). (**c**) Calcification rates calculated using the inorganic rate equation^28^ *G*=*k*(*Ω*−1)^*n*^, where *k* and *n* are the temperature-dependent constant and order of the reaction, respectively^28^. Because of opposing changes in pH_cf_ relative to DIC_cf_ (Fig. 1), *Ω*_cf_ and hence coral growth rates are strongly modulated reducing seasonal variations by twofold compared to those estimated from fixed condition experiments (G*). (**d**) Same as previous for *Porites* from Coral Bay (Ningaloo Reef, Western Australia).

Our findings also have major ramifications for the interpretation of the large number of experiments that have reported a strong sensitivity of coral calcification to increasing ocean acidification^32^. An inherent limitation of many of these experiments^33^ is that they were generally conducted under conditions of fixed seawater pH_sw_ and/or temperature, light, nutrients, and little water motion, hence conditions that are not conducive to reproducing the natural interactive effects between pH_cf_ and DIC_cf_ that we have documented here. A characteristic common to a variety of coral species grown under these artificial conditions is the apparently constant but limited sensitivity (one-third to one-half) of pH_cf_ relative to external changes in seawater pH_sw_ (refs 10, 17). While the reason for this apparently systematic but muted experimental response of pH_cf_ is still uncertain, it likely involves reduced and/or constant levels of metabolically produced DIC_cf_. Under such fixed conditions, we surmise that the supply of seawater DIC into the subcalicoblastic space (Fig. 1) becomes the dominant source and hence major influence on levels of DIC_cf_, with upregulation of pH_cf_ therefore acting as the major controller of *Ω*_cf_ and thereby affecting the perceived sensitivity of pH_cf_ to ocean acidification. This inference is supported by the fact that the observed pH_cf_ of *Porites* from both Davies and Ningaloo Reefs were closest to the pH_cf_ predicted from the constant condition experiments in winter when DIC_cf_ levels are naturally lowest due to reduced light and/or temperature, hence most similar to experimental predicted seawater end-member values. Clearly, since the interactive dynamics of pH_cf_ and DIC_cf_ upregulation do not appear to be properly simulated under the short-term conditions generally imposed by such artificial experiments, the relevance of their commonly reported finding of reduced coral calcification with reduced seawater pH must now be questioned.

In summary, we have now identified the key functional characteristics of chemically controlled calcification in reef-building coral. The seasonally varying supply of summer-enhanced metabolic DIC_cf_ is accompanied by dynamic out-of-phase upregulation of coral pH_cf_. These parameters acting together maintain elevated but near-constant levels of carbonate saturation state (*Ω*_cf_) of the coral’s calcifying fluid, the key driver of calcification. Although the maintenance of elevated but near-constant *Ω*_cf_ in mature coral colonies is not directly influenced by ocean acidification, it is however highly susceptible to thermal stress. In extreme cases of coral bleaching, the loss of endosymbionts disrupts the metabolic supply of DIC_cf_ as well as the metabolites necessary to operate the Ca-ATPase that upregulate pH_cf_ (refs 14, 34), thus effectively terminating calcification. So, although rising levels of *p*_CO_2__ can have adverse effects on the recruitment and growth of juvenile corals^35^,^36^,^37^,^38^, especially those lacking robust internal carbonate chemistry regulatory mechanisms, extreme thermal stress is detrimental to all symbiont-bearing corals^39^,^40^ regardless of their growth stage. We therefore conclude that the increasing frequency and intensity of coral bleaching events due to CO_2_-driven global warming constitutes the greatest immediate threat to the growth of shallow-water reef-building corals, rather than the closely associated process of ocean acidification.

## Methods

### Reef sites

*Porites* colonies were sampled from two reef systems: (1) Davies Reef (18.8° S, 147.63° E), a mid-shelf reef ∼100 km east-northeast of Townsville, Queensland, Australia in the central Great Barrier Reef, and (2) Coral Bay (23.19° S, 113.77° E), part of the Ningaloo Reef coastal fringing system of Western Australia. At Davies Reef, the annual range of daily average SST is 23–28.5 °C with a diurnal range of ∼0.5 °C or less^41^. *In situ* seawater temperature data extending back to 1987 for the core site at Davies Reef (18.83° S, 147.63° E) was compiled from a number of different temperature sensors deployed between a depth of ∼2 to ∼10 m maintained by the Australian Institute of Marine Science from October 1991 to December 2013 (http://data.aims.gov.au/aimsrtds/datatool.xhtml). To estimate seasonal changes in carbonate chemistry, we used the 24-h seawater carbonate chemistry data collected by Albright *et al*.^22^ on the lagoon side of the Davies Reef flat around the summer and winter extremes in both light and temperature. Their data showed that the daily average pH at that reef site was 8.02 in summer and 8.08 in winter; a seasonal range that was similar to seasonal minima and maxima observed and hind-cast at Coral Bay and hence similar to what would be expected from seasonal variations in temperature-driven *p*CO_2_ solubility. We therefore assumed that daily average pH at Davies Reef also followed seasonal changes in temperature according to pH_sw_=−0.010 × *T*+8.31.

At Coral Bay, SST generally ranges from 22–23 °C in winter to 27–28 °C in summer^21^. To hind-cast seasonal changes in reef-water temperature and pH, we first used time series of SST data from just offshore Coral Bay at ∼25 km resolution produced by Reynolds *et al*.^42^ before June 2010 and then at ∼1 km resolution produced by Chao *et al*.^43^ Both SST data products were then calibrated against *in situ* observations of temperature collected from a moored depth of ∼17 m as described by Falter *et al*.^21^ and previous model studies of wave-driven circulation. The carbonate chemistry of Coral Bay and offshore waters (∼2 km) were monitored between May 2011 and June 2012 and intermittently since then, with seasonal changes in offshore seawater pH_T_ (total scale) being found to be strongly correlated with seasonal changes in offshore temperature (pH_sw_=−0.012 × *T*+8.37, *r*^2^=0.86, *n*=13). To determine seasonal changes in pH at the back-reef site where the coral cores were recovered, the offshore pH was adjusted to account for the deviation in temperature due to local heating and cooling (see above), as well as the daily average decrease in total alkalinity of ∼10 μmol kg^−1^ at back-reef sites observed from measurements^44^.

### Boron isotopic pH proxy

Changes in the isotopic ratio of ^11^B (∼80%) and ^10^B (∼20%) are expressed in delta notation (in per mil, ‰) as:


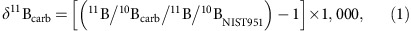


where ^11^B/^10^B_carb_ is the isotopic ratio measured in the coral carbonate and ^11^B/^10^B_NIST951_ is the isotopic ratio of the NIST SRM 951 boric acid standard. In seawater, boron exists as two different species, boric acid (B(OH)_3_) and the borate ion (B(OH)_4_^−^), with their relative abundance being pH dependent. The sensitivity of the δ^11^B proxy to the calcifying fluid pH_cf_ arises from the incorporation of only the borate ion species into the aragonite structure^45^,^46^,^47^, with the δ^11^B isotopic composition reflecting the pH sensitivity of the borate versus boric acid speciation. The pH of the calcifying fluid (pH_cf_) can thus be calculated from the δ^11^B measured in the coral carbonate (δ^11^B_carb_). The equation used to convert the δ^11^B_carb_ isotopic composition measured in the coral carbonate skeleton to a pH of the calcifying fluid (pH_cf_) is given by^48^:


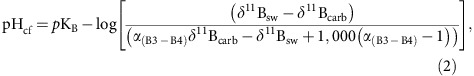


where δ^11^B_sw_ represents the δ^11^B in seawater (δ^11^B_sw_=39.61‰)^49^ and *α*_(B3−B4)_=1.0272 (ref. 50). The dissociation constant of boric acid *pK*_B_ has a well-established value of 8.597 at 25 °C and a salinity of 35 (ref. 51). Here we also assume that the calcifying fluid has the same δ^11^B composition as seawater since that is the ultimate source of boron and, due to the low *K*_D_ of B/Ca (ref. 19), the boron composition and concentration of the calcifying fluid remains essentially constant during calcification. Recent studies utilizing the δ^11^B pH_cf_ proxy as well as direct measurements of calcifying fluid pH using pH-sensitive dyes^9^,^18^, have also confirmed that under highly controlled artificial conditions of constant pH and temperature, corals upregulate the pH_cf_ of their calcifying fluid by ^1^/_3_ to ½ relative to ambient seawater pH.

### B/Ca constraints on calcifying fluid DIC concentrations

Prior studies indicate that borate rather than boric acid is the predominant species occupying the lattice position normally taken up by the carbonate ion^52^ in calcifiers that precipitate aragonite skeletons. Although there are a number of reaction pathways through which this substitution could occur^19^,^20^, it is likely to involve de-protonation of the borate species to create a divalent base ion with the same charge as that of the carbonate ion species (−2), to preserve the charge neutrality of the growing crystal:


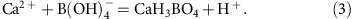


The partitioning of borate versus carbonate into aragonite is thus likely to be sensitive to solution pH^10^,^19^,^20^. Here the relevant partition coefficient *K*_D_ relating the molar ratio of 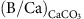 to the concentrations of the carbonate 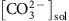 and borate 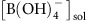 species in the precipitating solution is determined using:


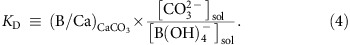


**Figure 5:**
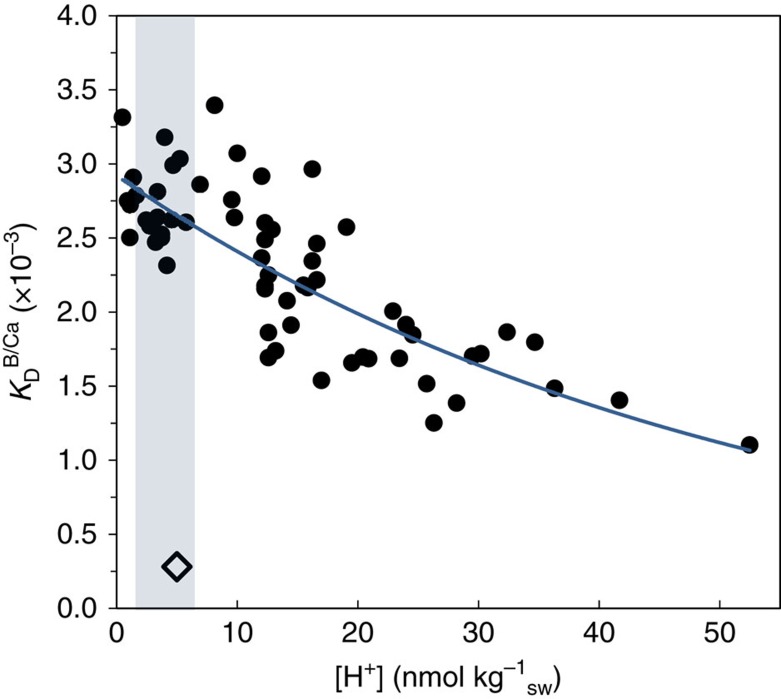
Experimentally determined B/Ca partition coefficient as a function of hydrogen ion concentration. Measured B/Ca partition coefficient (*K*_D_) as defined by equation (4) from the data of Holcomb *et al*.^19^ The line represents a best-fit exponential curve to the data with *K*_D_^B/Ca^=*K*_D,0_ exp(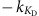
[H^+^]_T_), where *K*_D,0_=2.97±0.17 × 10^−3^ (±95% CI), 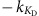
=0.0202±0.042, *r*^2^=0.64 and *n*=63. The range for pH_cf_ of upregulating calcifiers (that is, *Porites* spp.) is between ∼8 and ∼9 (shaded); equivalent to [H^+^]_T_ of between 1 and 10 nmol kg^−1^ giving a range in *K*_D_^B/Ca^ (× 10^−3^) of 2.6–2.8, and therefore relatively in-sensitive to changes in coral pH_cf_. Importantly, our experimentally determined *K*_D_^B/Ca^ value is an order of magnitude higher than the previous estimate by Allison *et al*.,^20^ (open diamond) and consistent with the substitution of B(OH)_4_^−^ with CO_3_^2−^.

Holcomb *et al*.^19^ conducted experiments quantifying the ratio of boron to calcium in aragonite precipitated inorganically under a wide range of carbonate chemistries (including pH) and total DIC and boron concentrations, as well as conditions of pH and DIC appropriate to those in the calcifying fluid of corals. Furthermore, Holcomb *et al*.^19^ also showed the close relationships between B/Ca, CO_3_^2−^ and *K*_D_ based on substitution reactions between B(OH)_4_^−^ and CO_3_^2−^. Re-analysing the Holcomb *et al*.^19^ data, we find (Fig. 5) that the observed *K*_D_ as defined in equation (4) shows the expected decrease as a function of the concentration of total active protons within the precipitating solution.

Thus, using the definition of *K*_D_ from equation (4) and its dependency on pH_cf_ as given by the inorganic data of Holcomb *et al*.^19^, we can now calculate the concentration of carbonate ions within the calcifying fluid (that is, [CO_3_^2−^]_cf_ from measurements of (B/Ca)_carb_ and pH_cf_, the latter derived from the skeletal boron isotopic ratio (δ^11^B_carb_). We further assume that [B_T_]_cf_ is equal to the total concentration of boron of ambient seawater and only a function of seawater salinity ([B_T_]_cf=_[B_T_]_sw_ at salinity=35). We therefore have:


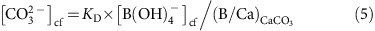


Where *K*_D_=0.00297exp(−0.0202 [H^+^]_T_ and for typical calcifying fluid pH_cf_ values *K*_D_ ∼0.0027, an order of magnitude higher than a previous estimate^20^. The concentration of DIC within the calcifying fluid is then calculated from the measured pH_cf_ (equation 1) and 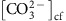 (equation 2) values using the programme CO_2_SYS provided by Lewis and Wallace^53^, with the carbonate species dissociation constants of Mehrbach *et al*.^54^ as re-fitted by Dickson and Millero^55^, the borate and sulfate dissociation constants of Dickson^51^,^56^, and the aragonite solubility constants of Mucci^57^. We also note that our use of a reliable experimentally determined *K*_D_ is now consistent with substitution of borate with carbonate ion, rather than the previously inferred^20^ substitution with bicarbonate ion, the latter assumption effectively negating the role of carbonate saturation state on calcification.

### Data availability

The coral geochemical and seawater carbonate chemistry and temperature data are available in Supplementary Data.

## Additional information

**How to cite this article:** McCulloch, M. T. *et al*. Coral calcification in a changing World and the interactive dynamics of pH and DIC upregulation. *Nat. Commun.* **8**, 15686 doi: 10.1038/ncomms15686 (2017).

**Publisher’s note**: Springer Nature remains neutral with regard to jurisdictional claims in published maps and institutional affiliations.

## Supplementary Material

Supplementary Data 1 Seawater (blue) and coral calcifying fluid parameters (orange). DIC = Dissolved Inorganic Carbon. pH*cf and G* are expected calcifying fluid (cf) pH and calcification rates (G) from fixed condition experimental calibrations (pH*cf = 0.32pHsw + 5.2). See manuscript for more details.

Peer Review File

## References

[b1] Darwin C. The Voyage of the Beagle: Journal of Researches into the Natural History and Geology of the Countries Visited During the Voyage of HMS Beagle Round the World Modern Library (2010).

[b2] Hughes T. P. . Climate change, human impacts, and the resilience of coral reefs. Science 301, 929–933 (2003). 12920289 10.1126/science.1085046

[b3] Hoegh-Guldberg O. Climate change, coral bleaching and the future of the world’s coral reefs. Mar. Freshw. Res. 50, 839–866 (1999).

[b4] Ciais P. . in *Climate Change 2013: The Physical Science Basis. Contribution of Working Group I to the Fifth Assessment Report of the Intergovernmental Panel on Climate Change* (eds Stocker, T. F. .) 465–570 (Cambridge University Press, 2014).

[b5] Furla P., Galgani I., Durand I. & Allemand D. Sources and mechanisms of inorganic carbon transport for coral calcification and photosynthesis. J. Exp. Biol. 203, 3445–3457 (2000). 11044383 10.1242/jeb.203.22.3445

[b6] Allemand D., Tambutté E., Zoccola D. & Tambutté S. in *Coral Reefs: An Ecosystem In Transition* Vol. III (eds Dubinsky, Z. & Stambler, N.) 119–150 (Springer, 2011).

[b7] Zoccola D. . Bicarbonate transporters in corals point towards a key step in the evolution of cnidarian calcification. Sci. Rep. 5, 9983 (2015). 26040894 10.1038/srep09983 PMC4650655

[b8] Al-Horani F. A., Al-Moghrabi S. M. & de Beer D. The mechanism of calcification and its relation to photosynthesis and respiration in the scleractinian coral *Galaxea fascicularis*. Mar. Biol. 142, 419–426 (2003).

[b9] Venn A. A. . Impact of seawater acidification on pH at the tissue-skeleton interface and calcification in reef corals. Proc. Natl Acad. Sci. USA 110, 1634–1639 (2013). 23277567 10.1073/pnas.1216153110 PMC3562847

[b10] Trotter J. A. . Quantifying the pH ‘vital effect’ in the temperate zooxanthellate coral *Cladocora caespitosa*: validation of the boron seawater pH proxy. Earth Planet. Sci. Lett. 303, 163–173 (2011).

[b11] Cohen A. L. & McConnaughey T. in *Biomineralization. Reviews in* *Mineralogy & Geochemistry*, Vol. 54 (eds Dove, P. M., Weiner, S. & Yoreo, J. J.) Ch. 6, 151–187 (The Mineralogical Society of America, 2003).

[b12] Yonge C. M., Nicholls A. G. & Yonge M. J. in *Studies on the Physiology of Corals*, Vol. 1 (British Museum, 1931).

[b13] Goreau T. Coral skeletal chemistry: physiological and environmental regulation of stable isotopes and trace metals in Montastrea annularis. Proc. R. Soc. Lond. B Biol. Sci. 196, 291–315 (1977).

[b14] Allemand D. . Biomineralisation in reef-building corals: from molecular mechanisms to environmental control. C. R. Palevol. 3, 453–467 (2004).

[b15] Tambutté S. . Coral biomineralization: from the gene to the environment. J. Exp. Mar. Biol. Ecol. 408, 58–78 (2011).

[b16] Cai W.-J. . Microelectrode characterization of coral daytime interior pH and carbonate chemistry. Nat. Commun. 7, 11144 (2016). 27041668 10.1038/ncomms11144 PMC4821998

[b17] McCulloch M. T., Trotter J. A., Falter J. & Montagna P. Coral resilience to ocean acidification and global warming through pH up-regulation. Nat. Clim. Chang. 2, 623–627 (2012).

[b18] Holcomb M. . Coral calcifying fluid pH dictates response to ocean acidification. Sci. Rep. 4, 5207–5211 (2014). 24903088 10.1038/srep05207 PMC4047535

[b19] Holcomb M., DeCarlo T. M., Gaetani G. A. & McCulloch M. Factors affecting B/Ca ratios in synthetic aragonite. Chem. Geol. 437, 67–76 (2016).

[b20] Allison N., Cohen I., Finch A. A., Erez J. & Tudhope A. W. Corals concentrate dissolved inorganic carbon to facilitate calcification. Nat. Commun. 5, 5741 (2014). 25531981 10.1038/ncomms6741

[b21] Falter J. L. . Assessing the drivers of spatial variation in thermal forcing across a nearshore reef system and implications for coral bleaching. Limnol. Oceanogr. 59, 1241–1255 (2014).

[b22] Albright R., Langdon C. & Anthony K. Dynamics of seawater carbonate chemistry, production, and calcification of a coral reef flat, central Great Barrier Reef. Biogeosciences 10, 6747–6758 (2013).

[b23] McCulloch M. T., Gagan M. K., Mortimer G. E., Chivas A. R. & Isdale P. J. A high-resolution Sr/Ca and δ^18^O coral record from the Great Barrier Reef, Australia, and the 1982-1983 El Niño. Geochim. Cosmochim. Acta 58, 2747–2754 (1994).

[b24] Falter J. L., Lowe R. J., Zhang Z. L. & McCulloch M. Physical and Biological controls on the carbonate chemistry of coral reef waters: effects of metabolism, wave forcing, sea level, and geomorphology. PLoS ONE 8, e53303 (2013). 23326411 10.1371/journal.pone.0053303 PMC3541250

[b25] Takahashi T. . Climatological distributions of pH, pCO_2_, total CO_2_, alkalinity, and CaCO_3_ saturation in the global surface ocean, and temporal changes at selected locations. Mar. Chem. 164, 95–125 (2014).

[b26] Holcomb M., Cohen A. L., Gabitov R. I. & Hutter J. L. Compositional and morphological features of aragonite precipitated experimentally from seawater and biogenically by corals. Geochim. Cosmochim. Acta 73, 4166–4179 (2009).

[b27] Fine M. & Tchernov D. Scleractinian coral species survive and recover from decalcification. Science 315, 1811 (2007). 17395821 10.1126/science.1137094

[b28] Burton E. A. & Walter L. M. Relative precipitation rates of aragonite and Mg calcite from seawater: Temperature or carbonate ion control? Geology 15, 111–114 (1987).

[b29] Erez J., Reynaud S., Silverman J., Scheinder K. & Allemand D. in *Coral* *Reefs*: *an ecosystem in transition* (eds Dubinsky, Z. & Stambler, N.) 151–176 (Springer, 2011).

[b30] Georgiou L. . pH homeostasis during coral calcification in a free ocean CO_2_ enrichment (FOCE) experiment, Heron Island reef flat, Great Barrier Reef. Proc. Natl Acad. Sci. 112, 13219–13224 (2015). 26438833 10.1073/pnas.1505586112 PMC4629382

[b31] McCulloch M. T. . Resilience of cold-water scleractinian corals to ocean acidification: Boron isotopic systematics of pH and saturation state up-regulation. Geochim. Cosmochim. Acta 87, 21–34 (2012).

[b32] Chan N. & Connolly S. R. Sensitivity of coral calcification to ocean acidification: a meta‐analysis. Glob. Chang. Biol. 19, 282–290 (2013). 23504739 10.1111/gcb.12011

[b33] Gattuso J. P. . Free-ocean CO_2_ enrichment (FOCE) systems: present status and future developments. Biogeosciences 11, 4057–4075 (2014).

[b34] Moya A. . Carbonic anhydrase in the scleractinian coral *Stylophora pistillata* characterization, localization, and role in biomineralization. J. Biol. Chem. 283, 25475–25484 (2008). 18617510 10.1074/jbc.M804726200

[b35] Tambutté E. . Morphological plasticity of the coral skeleton under CO_2_-driven seawater acidification. Nat. Commun. 6, 7368 (2015). 26067341 10.1038/ncomms8368 PMC4490415

[b36] Foster T., Falter J. L., McCulloch M. T. & Clode P. L. Ocean acidification causes structural deformities in juvenile coral skeletons. Sci. Adv. 2, e1501130 (2016). 26989776 10.1126/sciadv.1501130 PMC4788479

[b37] de Putron S. J., McCorkle D. C., Cohen A. L. & Dillon A. The impact of seawater saturation state and bicarbonate ion concentration on calcification by new recruits of two Atlantic corals. Coral Reefs 30, 321–328 (2011).

[b38] Albright R. & Langdon C. Ocean acidification impacts multiple early life history processes of the Caribbean coral *Porites astreoides*. Glob. Chang. Biol. 17, 2478–2487 (2011).

[b39] Randall C. J. & Szmant A. M. Elevated temperature affects development, survivorship, and settlement of the elkhorn coral, *Acropora palmata* (Lamarck 1816). Biol. Bull. 217, 269–282 (2009). 20040751 10.1086/BBLv217n3p269

[b40] Chua C. M., Leggat W., Moya A. & Baird A. H. Temperature affects the early life history stages of corals more than near future ocean acidification. Mar. Ecol. Prog. Ser. 475, 85–92 (2013).

[b41] Alibert C. & McCulloch M. T. Strontium/calcium ratios in modern *Porites* corals from the Great Barrier Reef as a proxy for sea surface temperature: Calibration of the thermometer and monitoring of ENSO. Paleoceanography 12, 345–363 (1997).

[b42] Reynolds R. W. . Daily high-resolution-blended analyses for sea surface temperature. J. Clim. 20, 5473–5496 (2007).

[b43] Chao Y., Li Z., Farrara J. D. & Hung P. Blending sea surface temperatures from multiple satellites and *in situ* observations for coastal oceans. J. Atmos. Ocean. Technol. 26, 1415–1426 (2009).

[b44] Zhang Z., Falter J., Lowe R. & Ivey G. The combined influence of hydrodynamic forcing and calcification on the spatial distribution of alkalinity in a coral reef system. J. Geophys. Res. Oceans 117, C04034 (2012).

[b45] Vengosh A., Kolodny Y., Starinsky A., Chivas A. R. & McCulloch M. T. Coprecipitation and isotopic fractionation of boron in modern biogenic carbonates. Geochim. Cosmochim. Acta 55, 2901–2910 (1991).

[b46] Mavromatis V., Montouillout V., Noireaux J., Gaillardet J. & Schott J. Characterization of boron incorporation and speciation in calcite and aragonite from co-precipitation experiments under controlled pH, temperature and precipitation rate. Geochim. Cosmochim. Acta 150, 299–313 (2015).

[b47] Hemming N. G. & Hanson G. N. Boron isotopic composition and concentration in modern marine carbonates. Geochim. Cosmochim. Acta 56, 537–543 (1992).

[b48] Zeebe R. & Wolf-Gladow D. A. in *Elsevier Oceanography Series*, vol. 65 (Elsevier, 2001).

[b49] Foster G. L., Pogge von Strandmann P. A. E. & Rae J. W. B. Boron and magnesium isotopic composition of seawater. Geochem. Geophys. Geosyst. 11, Q08015 (2010).

[b50] Klochko K., Kaufman A. J., Yoa W., Byrne R. H. & Tossell J. A. Experimental measurement of boron isotope fractionation in seawater. Earth Planet. Sci. Lett. 248, 261–270 (2006).

[b51] Dickson A. G. Thermodynamics of the dissociation of boric acid in synthetic seawater from 273.15 to 318.15 K. Deep Sea Res. A 37, 755–766 (1990).

[b52] Sen S., Stebbins J. F., Hemming N. G. & Ghosh B. Coordination environments of B impurities in calcite and aragonite polymorphs: a ^11^B MAS NMR study. Am. Mineral. 79, 819–825 (1994).

[b53] Lewis E. & Wallace D. *Program Developed for CO_2_ System Calculations* (Carbon Dioxide Information Analysis Center, Oak Ridge National Laboratory, U.S. Department of Energy, 1998).

[b54] Mehrbach C., Culberson C. H., Hawley J. E. & Pytkowicz R. N. Measurement of the apparent dissociation constants of carbonic acid in seawater at atmospheric pressure. Limnol. Oceanogr. 18, 897–907 (1973).

[b55] Dickson A. G. & Millero F. J. A comparison of the equilibrium constants for the dissociation of carbonic acid in seawater media. Deep Sea Res. 34, 1733–1743 (1987).

[b56] Dickson A. G. Standard potential of the reaction: AgCl(s)+1/2H_2_(g)=Ag(s)+HCl(aq) and the standard acidity constant of the ion HSO_4_^−^ in synthetic seawater from 273.15 to 318.15 K. J. Chem. Thermodyn. 22, 113–127 (1990).

[b57] Mucci A. The solubility of calcite and aragonite in seawater at various salinities, temperatures, and one atmospheric total pressure. Am. J. Sci. 283, 781–799 (1985).

